# Effect of foliar application of nano-nutrients solution on growth and biochemical attributes of tomato *(Solanum lycopersicum)* under drought stress

**DOI:** 10.3389/fpls.2022.1066790

**Published:** 2023-01-04

**Authors:** Areesha Mubashir, Zaib-un- Nisa, Anis Ali Shah, Munazza Kiran, Iqtidar Hussain, Naila Ali, Lixin Zhang, Mahmoud M. Y. Madnay, Waleed A. Alsiary, Shereen Magdy Korany, Muhammad Ashraf, Bandar A. Al-Mur, Hamada AbdElgawad

**Affiliations:** ^1^Institute of Molecular Biology and Biotechnology, The University of Lahore, Lahore, Pakistan; ^2^Department of Botany, Division of Science and Technology, University of Education, Lahore, Pakistan; ^3^Department of Agronomy, Faculty of Agriculture, Gomal University, Dera Ismail Khan, KP, Pakistan; ^4^College of Life Sciences, North West Agricultural and Forestry University, Xianyang, Shaanxi, China; ^5^Department of Botany and Microbiology, Faculty of Science, Cairo University, Giza, Egypt; ^6^Department of Environmental Sciences, Faculty of Meteorology, Environment and Arid Land Agriculture, King Abdulaziz University, Jeddah, Saudi Arabia; ^7^Department of Biology, College of Science, Princess Nourah bint Abdulrahman University, Riyadh, Saudi Arabia; ^8^Department of Botany and Microbiology, Faculty of Science, Beni-Suef University, Beni-Suef, Egypt

**Keywords:** nanoparticles, nutrients, drought, stress, tomato

## Abstract

**Introduction:**

Drought stress has drastically hampered the growth and yield of many crops. Therefore, environmentally safe agricultural techniques are needed to mitigate drought stress impact. To this end, foliar spray of nano-nutrients solution to (NNS) alleviate harmful aspects of drought stress.

**Methods:**

In a completely randomized design (CRD) experiment, seedlings were transplanted into pots at 2-3 leaf stage, each filled with loam-compost- organic manure soil (3:1:1). Plants were divided into two groups. (a) control group (b) applied stress group. Plants at vegetative stage were treated with 100% FC for control group and 60% FC for drought group, and these levels were maintained until harvesting. Three treatments of NNS with four levels i.e., 0%, 1%, 3% and 5% were given to all the pots after two weeks of drought stress treatment with a gap of 5 days at vegetative stage.

**Results and discussion:**

Application of 1% of nano-nutrient solution displayed an improvement in shoot length, shoot fresh and dry weight, number of leaves and flowers. Leaf chlorophylls and carotenoids and total phenolics contents were found maximum while minimum electrolyte leakage was observed at 3% application compared to control. Further, 1% application of NNS increased the Leaf RWC%, total soluble sugars, flavonoids contents. 5% NNS application exhibited higher total free amino acids with minimum lipid peroxidation rate in leaves of tomato under drought. Antioxidant enzyme activities increased in a concentration dependent manner as gradual increase was observed at 1%, 3% and 5%, respectively. Overall, this study introduced a new insights on using nano-nutrient solutions to maintain natural resources and ensure agricultural sustainability

## Introduction

Drought stress is a worldwide issue, and rapid climate change has increased the problem. These climatic changes are leading to increased occurrence and duration of drought episodes with concurrent reduction in crop yields (Ahanger et al., 2014). Drought alters physiological characteristics of plant leaves, such as lowering leaf photosynthetic and transpiration rates, enzyme activity, uptake of water and minerals and stomatal conductance, thus restraining crop productivity and yield (Ahanger et al., 2021). Drought stress results in the excess generation of ROS imparting oxidative damage to plants and hence the functioning of key metabolic pathways like photosynthesis, mineral uptake and assimilation is altered (Begum et al., 2020). Plants up-regulate antioxidant system and accumulation of osmolytes for neutralizing the excess ROS so that metabolism is protected (Ahanger and Agarwal, 2017; Begum et al., 2020).

Introduction of modern agricultural techniques like excessive chemical usage for improving agricultural productivity has rendered most of agricultural fields unproductive (Salehi et al., 2016). Biochar is a carbon-rich byproduct of oxygen-starved burning in low temperature (also referred as pyrolysis) of carbon - containing biomass such residue of crops, bedding of stall, cull lumber and sawmill wastes (Lehmann and Joseph, 2015). It plays many positive roles in agricultural production such as in composting improvement, in mitigation of salinity and drought stress as well as in improving crop production by ROS scavenging (Agegnehu et al., 2017). Therefore, use of nanobiochar can be an ideal choice for improving crop growth and yield in areas with low soil fertility and frequent stress outbreaks (Mahmud et al., 2020). Recent advancements in manufacturing techniques have led to fabrication of nano- materials ranging in size and shape, making base for the further advancement for specific application. Nanomaterials in biochar have received much recent attention among engineered biochars owing to its useful chemical and physical properties (Ramanayaka et al., 2020). Nanomaterials enter plant either through apoplast or symplast and can impart either positive or negative effect (Perez-de-Luque, 2017). Carbon nanoparticles are more useful to plants than their counter macro particles because of the greater surface area and more micro-porosity (Ramanayaka et al., 2020). Shekhawat et al. (2021) elucidated the role of engineered carbon nano-particles in promoting the growth and metabolism of *Vigna radiata*.

Tomato (*Solanum lycopersicum*) belongs to the nightshade family solanaceae, the third most economically important after grasses and legumes, and the most valuable in terms of vegetable crops (Foolad, 2007). Tomato contributes to a healthy and well-balanced diet and also plays a pivotal role in improving nutrition resources of poor population as compared to meat, milk, fruits and other high priced fruit items. It is a major contributor of antioxidants such as carotenoids (especially, lycopene and β-carotene), phenolics, ascorbic acid (vitamin C) and small amounts of vitamin E in daily diets (Rai et al., 2012). Current open pollinated varieties of tomato are unable to meet the domestic demand due to their low genetic potential, susceptibility to biotic and abiotic stresses, limited area under cultivation, water shortage and competition with major crops (Saleem et al., 2013).

Despite the documented significance of carbon-based nanomaterials in plant growth and development, the knowledge of the impact of carbon nanoparticles (CNPs) in the form of nano-nutrient solution on physiological and biochemical responses of vegetables is still scarce. Therefore, in this study we tested the validity of the following hypothesis: Foliar spray of nano-nutrients solution could alleviate harmful aspects of drought stress by improving some of the plant metabolites and antioxidant enzymes.

## Materials and methods

### Experimental design, treatments and sampling

A pot experiment was conducted in September-December 2020 at the green house University of Lahore, Lahore, Pakistan. Tomato seedlings were obtained from Vegetables Research Institute, VRI Faisalabad, Pakistan. Nano-nutrient solution was obtained from Shaanxi Dainong Huitai Biological Health Agricultural Technology Co., Ltd. Seedlings at 2-3 leaf stage were transplanted into plastic pots containing 20 kg loam-compost-organic manure soil (3:1:1). This experiment was designed in a completely randomized design (CRD) with factorial arrangement of four treatments and three replications in each treatment. Plants were grown till the fruit formation/yielding stage. Plants at vegetative stage were treated with two irrigation treatments (a) 100% FC for control group (b) 60% FC for drought group, and these levels were maintained until harvesting. Both the groups (control and drought stress) were foliar sprayed with nano-nutrients solution of biochar. Nano-nutrient solution was foliar applied three times with four levels i.e., 0%, 1%, 3% and 5% after two weeks of drought stress treatment with a gap of 5 days at vegetative stage.

Plant samples were collected after 20 days of foliar application of nano-nutrient solution for physiological and biochemical analysis. The growth and yield parameters were recorded at maturity.

### Plant biomass and growth parameters

Shoot and root lengths were measured manually using a scale, fresh weights of shoot and root and fruit, number of fruits and leaves was recorded immediately from each replication. However, samples were oven-dried individually at 60°C until the stable mass was obtained to record the results for dry weights of shoot and root.

### Leaf water status

Using the method of Barrs and Weatheley (1962), relative water content (RWC) was calculated. Fresh and mature leaves were weighed immediately for fresh weight, soaked in distilled water for 24 hours for turgid weight, oven dried for 24 hours at 80°C for dry weight and the given formula was used for the calculation of RWC.

### Measurement of photosynthetic pigments

Arnon’s method (1949) was used to examine the chlorophyll a, b, total chlorophyll, and carotenoids content in the fresh leaves of tomato kept under drought and control conditions by using the UV/V spectrophotometer HALO SB-10. The chlorophyll a and b contents, total chlorophyll contents, and carotenoid contents were calculated using formulas as Yoshida et al. (1976) reported, results were expressed as (mg/g FW).

### Biochemical parameters

After 3 weeks of nano-nutrients solution treatments, leaf samples were extracted in phosphate buffer solution (0.2 M) for biochemical analysis. According to Hamilton and Van Slyke, (1943) total free amino acids were calculated by adding 10% pyridine and 1% ninhydrin in 1 ml of leaf extract taken in 25 ml test tubes, kept at room temperature for 30 minutes and OD read at 570 nm. Following the method of Riazi et al. (1985) total soluble sugars were calculated. 0.5ml fresh leaves extract mixed with 2ml anthrone reagent (0.2% anthrone in 65% H_2_SO_4_ acid), heated for 10 minutes at 80°C in water bath, cooled for 30 minutes and measurements were taken at 620 nm using spectrophotometer.

### Leaf oxidative damage and electrolyte leakage

Lipid peroxidation was measured in terms of content of malondialdehyde (MDA) formation and was determined in accordance of Heath and Packer’s (1968) method. Briefly, fresh tissue was extracted in 1% TCA and extract was centrifuged at 10,000g for 5min. Supernatant was reacted with 0.5% thiobarbituric acid at 95°C for 30min. After cooling samples were centrifuged again at 5000g and optical density of supernatant was measured at 532 and 600 nm.

The percentage of electrolyte leakage (EL) in response to stress injury was determined by using the method of Lutts (1996). Leaves were cut in 1cm segments, washed with deionized water, kept in stoppered vials containing 10 ml deionized water and incubated at 25°C. Electrical conductivity of the bathing solution was determined after almost 3 hours. Samples were then autoclaved at 120°C for 20 minutes and second reading was obtained upon equilibration at 25°C. Calculations were done using the following formula:

EC % = (L1/L2) × 100

### Secondary metabolites

Total phenolic contents of leaves were estimated using the Folin-Denis reagent as described by Julkunen-Titto (1985). The 1ml leaf extracts (80% acetone) were mixed with 5ml Na_2_CO_3_ and 1ml FC regent put into solution. Solution was shaken with the help of shaker for 20 minutes. Absorbance of the resulting blue color was measured at 765 nm. For the determination of total flavonoid contents, 0.1ml ammonia was added in 0.1ml fresh leaf extract. The falcon tubes were shaken properly with the help of rotary shaker. Then 2 ml sulfuric acid (H_2_SO_4_) was added and the solution was left for color generation for about 30 minutes. Measurement was taken at 465 nm, as reported by Pekal and Pyrzynska (2014).

### Activity of antioxidant enzymes

Assay of catalase and peroxidase enzymes was carried according to the method of Chance and Maehly (1955) and change in absorbance was recorded at 240 nm for 3 minutes. Superoxide dismutase activity was measured using the method of Dhindsa et al. (1981) and optical density was recorded at

### Statistical analysis

The numerical data collected was evaluated by Analysis of Variance (ANOVA) using HSD. Significant differences among treatments were analyzed by Tukey’s test. The least significant difference was used to compare means at p≤.05.

## Results

### Growth attributes

Drought resulted in a progressive and significant decline in growth attributes (**Table 1A**) and fresh and dry biomass of tomato plants (**Table 1B**). Water deficit stress of 60% FC decreased the number of leaves by 19.4%, number of flowers by 13.6%, shoot length and root length by 11.1% and 10.7% respectively, fruit weight per plant was reduced by 18.3%, shoot fresh weight and root fresh weight by 31.8% and 14.6% respectively as compared to control plants. Similarly, the shoot dry weight and root dry weight was also declined by 24.4% and 22.9% respectively under drought conditions (60% FC) as compared to control plants without drought stress.

**Table 1A:** The effect of foliar application of nano-nutrient solution on growth attributes of tomato under drought stress.

Condition	Foliar (%)	shoot length (cm)	Root length (cm)	Number of leaves (n)	Number of flowers (n)	Fruit weight per plant (g)
Control	0 1 3 5	15 ± 0.5^c^ 20.6 ± 0.3^a^ 19.6 ± 0.3^ab^ 18 ± 0.5^b^	9.3 ± 0.3^abcd^ 9.6 ± 0.3^abc^ 10.6 ± 0.3^bcd^ 12.3 ± 0.3^ab^	12.3 ± 0.3^a^ 13.3 ± 0.3^cd^ 12 ± 0^bc^ 11 ± 0.5^cd^	7.3 ± 0.33^de^ 10.3 ± 0.33^a^ 8.6 ± 0.33^c^ 8.33 ± 0.33^cd^	8.1 ± 0.1^f^ 13.3 ± 0.3^ef^ 9.3 ± 0.3^de^ 10.6 ± 0.3^cd^
Drought	0 1 3 5	13.3 ± 0.3^a^ 21.3 ± 0. 3^a^ 18 ± 0.5^b^ 15 ± 0^c^	8.3 ± 0.3^d^ 10.3 ± 0.3^cd^ 11.6 ± 0.3^abc^ 12.6 ± 0.3^a^	11 ± 0.5^cd^ 11.6 ± 0.3^bc^ 9.3 ± 0.3^de^ 8.0 ± 0.5^e^	6.33 ± 0.33^f^ 9.66 ± 0.33^ab^ 7.6 ± 0.33^d^ 7.0 ± 0.00^ef^	8.3 ± 0.3^a^ 11 ± 0.5^b^ 12.6 ± 0.3^bc^ 13.3 ± 0.3^b^
Anova (F-Value)						
Drought (D) Foliar (F) Drought*Foliar		*** *** ***	* *** ns	*** *** *	ns *** ns	*** *** ***

Each value is a mean of three replicates ± standard errors; different alphabetic letters indicate significant differences (P ≤ 0.05) among treatments using Tukey’s test; * and *** indicate significant at p ≤ 0.05 and p ≤ 0.001 respectively; ns indicate non-significant difference.

**Table 1B:** The effect of foliar application of nano-nutrient solution on plant biomass under drought.

Condition	Foliar (%)	Shoot fresh weight (g)	Shoot dry weight (g)	Root fresh weight (g)	Root dry weight (g)
Control	0 1 3 5	23.2 ± 0.2^bc^ 25.3 ± 0.2^a^ 23.4 ± 1.01^bc^ 19.5 ± 0.5^de^	4.6 ± 0.1^ab^ 5.2 ± 0.2^a^ 4.6 ± 0.1^cd^ 3.9 ± 0.06^de^	3.6 ± 0.19^ab^ 4.6 ± 0.3^a^ 4.16 ± 0.1^bc^ 3.9 ± 0.05^bc^	1.24 ± 0.02^ab^ 1.49 ± 0.00^c^ 1.3 ± 0.08^d^ 1.25 ± 0.08^e^
Drought	0 1 3 5	15.8 ± 0.3^ab^ 20.6 ± 0.9^cd^ 19.4 ± 0.7^de^ 16.3 ± 0.3^e^	3.5 ± 0.19^bc^ 4.6 ± 0.2^cd^ 3.9 ± 0.15^de^ 3.5 ± 0.06^e^	5.5 ± 0.1^c^ 3.5 ± 0.1^c^ 5.1 ± 0.2^a^ 3.9 ± 0.05^bc^	2.15 ± 0.04^a^ 1.26 ± 0.03^e^ 1.93 ± 0.09^ab^ 1.49 ± 0.14^c^
Anova (F-Value)					
Drought (D) Foliar (F) Drought*Foliar		*** *** ***	ns *** *	*** *** **	** *** ***

Each value is a mean of three replicates ± standard errors; different alphabetic letters indicate significant differences (P ≤ 0.05) among treatments using Tukey’s test; *,**, and *** indicate significant at p ≤ 0.05, p ≤ 0.01and p ≤ 0.001 respectively; ns indicate non-significant difference.

The foliar application of NNS produced stimulatory effects on growth parameters under drought and control conditions. Specifically, the exogenously applied NNS at the concentration of 1% and 3% has more positive effect on growth attributes (number of leaves and flowers, plant length, plant fresh and dry biomass, fruit weight per plant etc.) as compared to the drought alone or even under non – stressed conditions. The foliar application of NNS at 1% concentration significantly increased the number of leaves by 20.6% and 11.1%, number of flowers by 52.6% and 40.9%, shoot length by 60% and 37.7%, shoot fresh weight by 30% and 9.1%, shoot dry weight by 32.3% and 13.5% as compared to control plants that received no NNS application, in both drought (60% FC) and control conditions respectively. However, 3% application increased root length by 36% and 14.2%, fruit weight per plant by 90% and 63.2%, root fresh weight by 56.8% and 33.8% and dry weight of root by 78.4% and 28.3% as compared to plants with no NNS treatment, in both drought and control conditions respectively.

### Relative water content (RWC)

**Figure 1:**
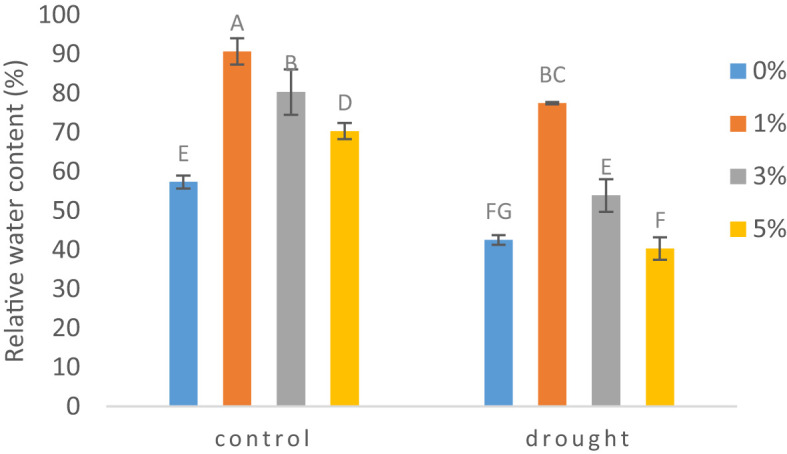
The effect of different concentrations of foliar application of nano-nutrients solution on relative water content of leaves of tomato *(Solanum lycopersicum)* under drought. Mean values are the average of three replicates. The upper case alphabetic letters indicate significant differences (P≤0.05) among treatments using Tukey's test.

Drought significantly influenced the leaf water status of tomato plant. A considerable reduction was seen by 26% in relative water content of leaves under drought as compared to control plants (**Figure 1**). On the other hand, foliarly applied nano-nutrients solution at 1% concentration increased the RWC by 45% and 37% as compared to non-treated plants under drought and control conditions respectively.

### Leaf pigments

**Figure 2:**
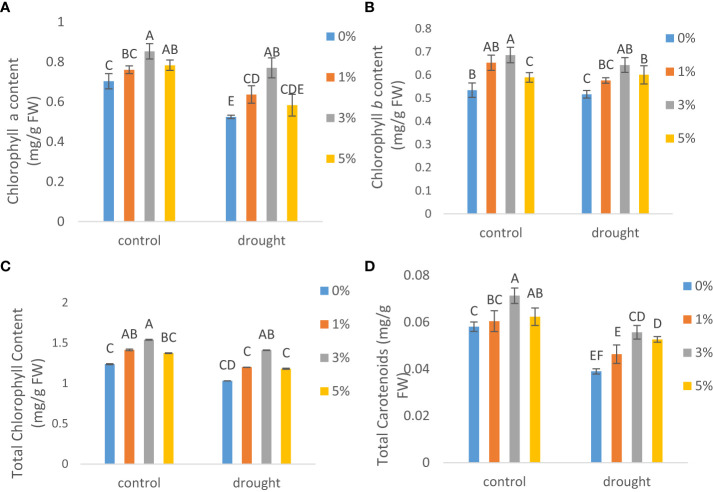
The effect of different concentrations of foliar application of nano-nutrients solution on **(A)** chlorophyll *a*, **(B)** chlorophyll *b*, **(C)** total chlorophyll and **(D)** carotenoid contents in leaves of tomato *(Solanum lycopersicum)* under drought. Mean values are the average of three replicates. The upper case alphabetic letters indicate significant differences (P≤0.05) among treatments using Tukey's test.

Leaf pigments of tomato were influenced significantly by drought. A considerable reduction was observed in chlorophyll a (**Figure 2A**) by 25.4%, chlorophyll b (**Figure 2B**) by 3.3%, total chlorophyll (**Figure 2C**) by 17% and in carotenoids content (**Figure 2D**) by 32.8% under drought conditions (60% FC) as compared to control plants. However, exogenous application of NNS in 3% concentration increased chlorophyll a by 46.7% and 21.3%, chlorophyll b by 24.5% and 28.4%, total chlorophyll by 26% and 19.2% and total carotenoids by 42.7% and 22.8% as compared to plants within NNS under drought and control conditions respectively.

### Biochemical analysis

**Figure 3:**
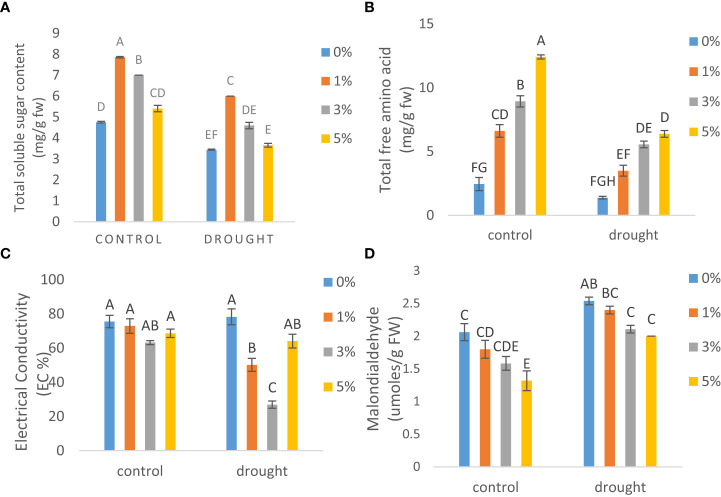
The effect of different concentrations of foliar application of nanonutrient solution on **(A)** total soluble sugars, **(B)** total free amino acids, **(C)** electrolyte leakage and **(D)** malondialdehyde in leaves of tomato (Solanum lycopersicum) under drought. Mean values are the average of three replicates. The upper case alphabetic letters indicate significant differences (P≤0.05) among treatments using Tukey's test

Significant reduction was observed by 28% in total solule sugars (**Figure 3A**) and by 43.5% in total free amino acids (**Figure 3B**) resectively, under drought as compared to control plants. However, an increasing trend for both soluble sugars and total free amino acids was seen by the foliar application of nano-nutrients solution. 1% treatment of nano-nutrientss maximized total soluble sugars by 2-fold and, 5% concentration increased total free amino acids by 4.5 and 5-fold under drought and control conditions respectively as compared to plants with no treatment of nano-nutrientss.

### Electrical conductivity and malondialdehyde content

Drought stress resulted in an increase in leakage of electrolytes (**Figure 3C**) and lipid peroxidation (**Figure 3D**) by 1-fold in leaves as compared to control. However, foliarly applied nano-nutrientss at 3% reduced EC (%) by 1- and 3-fold and 5% application reduced leaf oxidative damage by 1.5- and 1-fold as compared to non-treated plants under control and drought conditions respectively.

### Secondary metabolites

**Figure 4:**
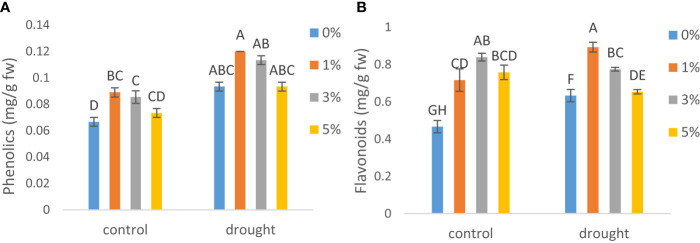
The effect of different concentrations of application of nano-nutrients solution on **(A)** phenolics and **(B)** flavonoids in leaves of tomato *(Solanum lycopersicum)* under drought. Mean values are the average of three replicates. The upper-case alphabetic letters indicate significant differences (P≤0.05) among treatments using Tukey's test.

In the present study, stress given in the form of drought increased the secondary metabolites (phenolics and flavonoids). Total phenolics (**Figure 4A**) were increased by 1.5- and flavonoids (**Figure 4B**) by 1-fold under drought as compared to control plants. Moreover, 1% exogenous application of nano-nutrients solution furthur increased the phenolics by 33% and 28% as compared to non-treated plants under control and drought respectively. However, flavonoid contents were increased by 79% by 3% application under control condition and 41% by the application of 1% treatment under drought.

### Antioxidant enzymes

**Figure 5:**
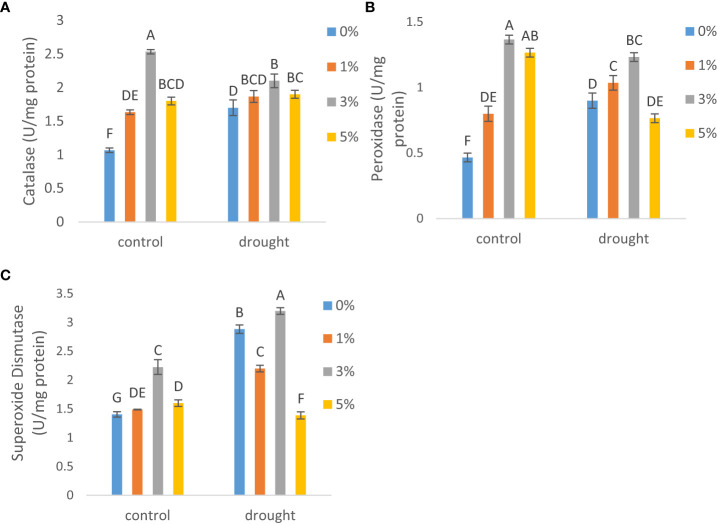
The effect of different concentrations of foliar application of nano-nutrients solution on antioxidant enzymes **(A)** catalase **(B)** peroxidase and **(C)** superoxide dismutase in leaves of tomato *(Solanum lycopersicum)* under drought. Mean values are the average of three replicates. The upper case alphabetic letters indicate significant differences (P≤0.05) among treatments using Tukey's test.

The antioxidative activities were observed to assess the effect of foliar spray of nano-nutrientss on antioxidants under drought. Under drought, CAT activity (**Figure 5A**) increased by 59%, POD activity (**Figure 5B**) by 92% and SOD activity (**Figure 5C**) by 2-fold than that of non-stressed condition. Meanwhile, the foliar application of nano-nutrients solution further increased the activities of CAT, POD and SOD under both stressed and non- stressed conditions. Specifically, 3% application increased CAT activity by 59% and 1.5- fold, POD activity by 3-and1-fold and SOD activity by 58% and 10% as compared to plants without any treatment of nano-nutrientss under control and drought conditions respectively.

## Discussion

Climatic changes around the world often make plants vulnerable towards various abiotic stresses which limit the crop productivity and yield. Similarly, drought stress has the most devastating effect on crops worldwide. In this backdrop effect exogenous application of foliar application of nano-nutrientss on morphological, physiological and biochemical parameters of drought stressed tomato by enhancing its drought tolerance was investigated.

Drought stress is one of the most harmful stresses which affect plant water metabolism and induce major morphological, physiological, and biochemical alterations in plants. In our study, it is clearly observed that drought has decreased many growth and yield attributes (e.g., number of leaves, flowers, fresh and dry biomass of roots and shoots as well as root and shoot length etc.). our results are consistent with previous reports in a variety of plants (Torres-Ruiz et al., 2015) and (Zhang et al., 2018). The findings indicated that from different concentration of nano-nutrients solution (1%, 3% and 5%) applied foliarly on tomato plants, 1% increased several growth parameters and fresh and dry biomass (number of leaves and flowers, shoot fresh and dry weight, shoot length), and few parameters (root length, dry weight and fruit weight per plant under drought) were improved by 5% application. Similarly, (Panda et al., 2020) discovered an increase in shoot length and number of leaves by foliar spraying a nano-based, water soluble foliar fertilizer on tomato plants. Seed priming with 400ppm nano ZnO particles or seed priming + foliar spray on tomato plants boosted yield and growth, according to Khanm et al. (2017). Nano phosphorus at 20 and 30 ppm increased root length and shoot length in cowpea, according to Priya et al. (2016). Plants treated to ZnO nanoparticles produced fruits which were larger and heavier than control plants (Bhardwaj et al., 2022).

Relative water content of plants has been seen greatly affected by drought. In the present study RWC decreased in water deficit condition and similar results has been reported in many previous studies (Xiao et al., 2009). The exogenous application of nano-nutrientss in varying concentrations gave positive results with improving the leaf water status of tomato plants. 1% application maximized RWC in tomato under drought and our results are consistent with previous reports. In both seasons, soaking the rice grains in silicon and selenium nanoparticles and applying selenium nanoparticles to the leaves resulted in the highest relative water content as stated by Badawy et al. (2021).

Drought stress reduced the production of total chlorophylls and carotenoids significantly and our work is in accordance with the previous findings as reported by Ahanger et al. (2021). Chlorophyll intermediates including δ-ALA are reduced in number by drought (Dalal and Tripathy, 2012). Results of the current study suggested that showed that the use of NNS improved the concentration of δ -ALA, which in turn improved chlorophyll and photosynthetic functions in tomato, as well as prevented drought-induced growth decline. Among them, 3% application has shown significantly higher chlorophyll *a* and chlorophyll *b* contents and a similar trend was seen for total chlorophyll and carotenoids where 3% NNS application has more positive effects under both drought and control conditions. Previously, Bhardwaj et al. (2022) reported that for increasing nanoparticle concentrations up to 500 mg kg^-1^, the chlorophyll content of tomato plants treated with aerosol-foliar sprayed TiO_2_ nanoparticles increased. In carrots, graphene oxide and zinc oxide NPs at 0.10 mg/ml were beneficial in enhancing chlorophyll and carotenoid content, reported by Siddiqui et al. (2019).

Water deficit stress also resulted in an increase in ROS generation, electrolyte leakage and lipid peroxidation which ultimately resulted in significant oxidative damage in leaves of tomato. Previously, several workers have also observed increased oxidative damage owing to drought (Ahanger and Agarwal, 2017; Mamnabi et al., 2020) which is similar to our findings. lypoxygenase and protease activity are associated with increased ROS generation, resulting in more damage to lipids and proteins. As a result, structural and functional changes in key macromolecules such as proteins and fatty acids are induced (Nahar et al., 2016). Nanomaterials have a cumulative effect on antioxidant metabolites such as phenols, flavonoids, osmolytes, and antioxidant enzymes which causes a decrease in the oxidative effects of ROS. (Sadak and Bakry, 2020; Hussain et al., 2020). This study also indicated drought-induced increase in lipoxygenase and protease activity which was greatly reduced by NNS supplementation, proclaiming that NNS plays a beneficial role in preserving proteins and lipids in tomato under such conditions. 3% application resulted in a decline in electrolyte leakage however 5% brought down the values for oxidative damage. Similarly, Khan et al. (2020) discovered that water-stressed *Vicia faba* L. plants had increased electrolyte leakage and lipid peroxidation and nano-TiO_2_ application to stressed plants, reduced them.

Moreover, as intimidated by our findings, other researchers (Ahanger and Agarwal, 2017; Begum et al., 2020) have also documented rise in antioxidant activity as a result of drought stress. Increased antioxidant enzyme activity has been shown to reduce drought-influenced ROS-induced photosynthesis reduction by enhancing membrane stability and removing ROS quickly (Yang et al., 2014). Nanomaterials on the other hand, increase antioxidant enzyme activity, resulting in lower ROS buildup and hence improved photosynthesis, growth and protection of essential metabolic pathways (Sharma et al., 2019). However, depending on the concentration of nanomaterial employed, the impact may vary significantly. Among antioxidant enzymes, SOD acts as a first line of defense against harmful radicals, removing superoxide from cells and preventing impairment of metabolic pathways such as photosynthesis. (Choudhury et al., 2017). Aside from that, CAT and POD are important antioxidant enzymes that help to neutralize H_2_O_2_. Our results demonstrated that 3% application of NNS maximized antioxidants including CAT, POD and SOD. Ahmad et al. (2020) recently demonstrated that under arsenic stress, the application of ZnO nanoparticles considerably up-regulated antioxidant functioning which lead to growth and photosynthetic modulations and Mehrian et al. (2015) stated that, antioxidant activity of SOD, CAT and POD enzymes increased in shoots and roots of tomato when treated with silver nanoparticles.

Furthermore, Increased phenol and flavonoid synthesis in NNS-sprayed tomato plants further improved the antioxidant system under control and drought conditions. By protruding into the lipid bilayer, both phenols and flavonoids have the ability to prevent ROS-mediated lipid peroxidation and maintain membrane fluidity and function (Oteiza et al., 2005). According to the findings of this report, enhanced secondary metabolite content and up-regulation of the antioxidant system in tomato plants foliarly treated with NNS protects photosynthesis by boosting the redox buffer components for effective electron transport. 1% treatment enhanced the activity of phenolics under both control and drought conditions and flavonoids under drought condition however the later was improved better by 5% application under control. Comparably, another study found that silver NMs elevated oxidative stress and increased the levels of phenolics and flavonoids in potato (Homaee and Ehsanpour, 2016) and (Genady et al., 2016) stated that exposure to copper sulphate nanoparticles resulted in an increase in the content of secondary metabolites (phenolics and flavonoids) in *Verbena bipinnatifida*.

In addition, exogenous application of NNS resulted in production of more osmolytes such as sugars and free amino acids, which may have contributed considerably to tissue water content maintenance. Justifying our results, Ahanger and Agarwal (2017) also reported an increase in in the accumulation of osmolytes under drought in different crops. The ability of osmolytes to sustain a water potential gradient for continuous water intake contributes to their protective effect during drought (Ahanger et al., 2014). More accumulation of suitable osmolytes has a major impact on plant development and yield performance, so in this investigation, NNS spraying increase in osmolytes may lead to improved tomato growth and yield production. 1% application improved content of total soluble sugars however total free amino acids were more liberated at 5% concentration. The use of nano-ZnO and compost on *Linum usitatissimum* has recently been shown to boost the accumulation of suitable osmolytes such as free amino acids and soluble sugars, resulting in increased growth and yield (Sadak and Bakry, 2020).

## Conclusion

Conclusively, growth, physiological and biochemical activities of tomato were rendered by the excessive generation of toxic ROS under drought. The growth and biomass of tomato plants was improved by the exogenous application of nano-nutrientss of nano-biochar and it also ameliorated oxidative stress generated by drought as it reduced ROS accumulation, lipid peroxidation and enhanced membrane stability. Up-regulated antioxidant enzymes, secondary metabolites, and osmolytes due to the foliar treatment of NNS justifies its positive role in averting drought mediated damage to tomato.

## Data availability statement

The original contributions presented in the study are included in the article/supplementary material. Further inquiries can be directed to the corresponding authors.

## Author contributions

AM, HA, AAK, MYM, SMK, Experimentation; Z-u-N, SMK, WAA, MS, HA, Supervision and Research Design; AS, NA, IH, HA, BAA, Review and Drafting; MK, HA, AAK, MYM, LZ, SMK, Validation and Statistical Analysis; IH, HA, AAK, Drafting and Validation. All authors contributed to the article and approved the submitted version.

## Funding

Princess Nourah bint Abdulrahman University Researchers Supporting Project number (PNURSP2023R214), Princess Nourah bint Abdulrahman University, Riyadh, Saudi Arabia.

## Acknowledgments

Princess Nourah bint Abdulrahman University Researchers Supporting Project number (PNURSP2023R214), Princess Nourah bint Abdulrahman University, Riyadh, Saudi Arabia.

## Conflict of interest

The authors declare that the research was conducted in the absence of any commercial or financial relationships that could be construed as a potential conflict of interest.

## Publisher’s note

All claims expressed in this article are solely those of the authors and do not necessarily represent those of their affiliated organizations, or those of the publisher, the editors and the reviewers. Any product that may be evaluated in this article, or claim that may be made by its manufacturer, is not guaranteed or endorsed by the publisher.
